# Genome Sequences of a Novel Recombinant Duck Circovirus in China

**DOI:** 10.1128/genomeA.01181-16

**Published:** 2016-10-20

**Authors:** Wenchao Sun, Min Zheng, Huihui Cao, Huijun Lu, Xiankai Wei, Yi Pan, Hongyun Zhang, Jiaoxiu Su, Jun Li, Ningyi Jin

**Affiliations:** a College of Animal Science and Technology, Guangxi University, Nanning, People’s Republic of China; b Institute of Military Veterinary, The Academy of Military Medical Sciences, Changchun, People’s Republic of China; c Guangxi Center for Animal Disease Control and Prevention, Nanning, People’s Republic of China; d Yulin Center for Animal Disease Control and Prevention, Yulin, People’s Republic of China

## Abstract

In this study, YN26/2013, a novel recombinant duck circovirus (DuCV), was isolated from a Muscovy duck in Yunnan Province, southern China. The whole genome of YN26/2013 consists of 1,987 nucleotides (nt), the same genomic size as that of the DuCV-2 genotype. However, YN26/2013 shares 91.5 to 94.3% nucleotide identity similarity with previously reported type I (DuCV-1) viruses. Importantly, a novel putative recombinant event between DuCV-1 and DuCV-2 was identified as occurring within the 987- to 1111-nt region of the YN26/2013 genome.

## GENOME ANNOUNCEMENT

Duck circovirus (DuCV), a tentative member of the genus *Circovirus* of the family Circoviridae, was first isolated in Germany in 2003 (1). Mallard, Muscovy, Mule, and Pekin ducks are susceptible to DuCV infection (2). The infected birds showed feathering disorders, growth retardation, and low weight (3). Histopathologic examination demonstrated lymphocyte depletion, necrosis, and histiocytosis in the bursa of Fabricius (4).

DuCV is a small (15 to 16 nm in diameter), round, nonenveloped, and single-stranded DNA virus having a circular genome of 1,987 to 1,996 nucleotides (nt) (5). The genome contains two major open reading frames (ORFs) encoding the replicase (V1) and capsid (C1) proteins, respectively (6, 7). There are two noncoding intergenic regions (IRs) between the 5′ and 3′ ends of the two major ORFs (8).

In this study, liver, lung, and spleen samples were collected from Muscovy ducklings age ~30 days from Yiliang County of Yunnan Province, China, for routine surveillance on a live bird market located in Baise City of Guangxi Province in April 2013. DNA was extracted using the QIAamp DNA minikit (Qiagen). Two overlapping fragments encompassing the entire viral genome were amplified by PCR using primer pairs. PCR products were cloned into pMD18-T cloning vector (TaKaRa, Dalian, China) and sequenced by Invitrogen (Guangzhou, China).

Our results indicated that the full genomic length of YN26/2013 is 1,987 nt, which corresponds with those of DuCV-2 isolates. Compared with other reported DuCV-1 and DuCV-2 genomes, the YN26/2013 genome shared 91.5 to 94.3% and 84.3 to 87.0% nucleotide sequence identities, respectively. To investigate the putative recombination event, the Recombination Detection Program version 4.0 (RDP4) and Simplot Program version 3.5.1 were used to identify putative parental sequences and to localize possible recombination breakpoints in YN26/2013. The recombination events revealed that YN26/2013 clustered with the minor parental isolate GX1104 in the recombinant regions 987 to 1111 nt but in the nonrecombinant regions of other DuCV-1 isolates.

In our study, the 5′-end IR, two major ORFs, and the first-copy repeat in the 3′-end IR of YN26/2013 showed the highest similarity with those of DuCV-1 isolates, whereas the other three-copy repeats in 3′-end IR were almost the same as those in DuCV-2 isolates. This strongly suggested intergenotypic recombination within this region.

In conclusion, we have sequenced the full-length genome of YN26/2013, a recombinant DuCV isolated from Muscovy duck in China and, for the first time to our knowledge, provided evidence of intergenotypic recombination events within 3′-intergenic region of the DuCV genome. Further study is needed to investigate its biological significance and mechanism.

### Accession number(s).

The GenBank accession number is KR491946.
